# *Phialophora chinensis* fungal keratitis: An initial case report and species identification

**DOI:** 10.1016/j.ajoc.2023.101800

**Published:** 2023-01-19

**Authors:** Briana Ply, Connie F. Cañete-Gibas, Carmita Sanders, Nathan P. Wiederhold, Rachel A. Dandar, John D. Sheppard, Albert Y. Cheung

**Affiliations:** a Department of Ophthalmology, Eastern Virginia Medical School, Norfolk, VA, USA; b Department of Pathology and Laboratory Medicine, University of Texas Health Science Center at San Antonio, San Antonio, TX, USA; c Virginia Eye Consultants/CVP Physicians, Norfolk, VA, USA

**Keywords:** Fungal keratitis, *Phialophora chinensis*, Corneal collagen crosslinking, Corneal ulcer, Perforation, OD, Right eye, OS, Left eye, HSV, herpes simplex virus, FTL, Fungus Testing Laboratory, CXL, corneal cross-linking, PK, Penetrating keratoplasty, GHJ, graft-host-junction, PFA, potato flakes agar, OA, oatmeal agar, MIC, Minimum inhibitory concentrations, RFLP, restriction fragment length polymorphisms

## Abstract

**Purpose:**

To report the initial case of microbial keratitis caused by *Phialophora chinensis*, a rare cause of fungal keratitis.

**Observations:**

A 66-year-old gentleman with a complex right eye (OD) ocular history including herpes simplex virus infectious epithelial keratitis with subsequent neurotrophic keratopathy, and prior combined *Candida albicans* and *parapsilosis* fungal keratitis presented with pain OD in the absence of an antecedent trauma. The patient was found to have a filamentous fungal keratitis, which was subsequently cultured and identified as *Phialophora chinensis* by the laboratory. Despite topical and oral antifungal treatment based on sensitivities determined by the lab, the patient ultimately required intrastromal and subconjunctival antifungal injections, corneal crosslinking, and superficial keratectomy with amniotic membrane to clinically improve. The fungal keratitis recurred twice, with each occurrence rapidly progressing to corneal perforation. Months after the second penetrating keratoplasty, the patient's mental status declined due to multiorgan failure. An occult pulmonary malignancy was discovered during this hospital stay, and the patient was lost to follow-up after entering hospice.

**Conclusions and Importance:**

We report a unique case of fungal keratitis caused by *Phialophora chinensis* and the subsequent management, including both medical and surgical interventions*.* Despite a multimodal treatment regimen, this case demonstrates the recalcitrant and potentially recurrent nature of fungal keratitis caused by *P. chinensis*.

## Highlights

• *Phialophora chinensis* is an uncommon cause of fungal keratitis.
• The course may be aggressive, chronic, and potentially lead to corneal perforation.
• A multimodal treatment regimen may be necessary.
• Our case shows the recalcitrant and potentially recurrent nature of *P. chinensis.*

## 1 Introduction

*Phialophora* are uncommon causes of ocular infection, despite being common causes of chromoblastomycosis. These dematiaceous fungi are characterized by dark brown, thick-walled sclerotic bodies (muriform cells) in infected tissues. *Phialophora* are commonly found in tropical areas, and infection most commonly results from a traumatic inoculation.^1^ In addition to causing chronic cutaneous and subcutaneous tissue infection, *Phialophora* have been implicated in mycotic keratitis^2^, ^3^, ^4^, ^5^, ^6^ and endophthalmitis.^7^^,^^8^ Nearly all *Phialophora* ocular infections have been attributed to *P. verrucosa.*

In our patient, *Phialophora chinensis* was cultured and identified by the laboratory. Although closely related to *P. verrucosa, P. chinensis* was recognized as a separate species in 2017 by Li et al.^9^ These species can differ significantly in terms of pathogenicity and virulence. We report the initial case of *P. chinensis* fungal keratitis, describe our species identification method, and discuss management using a multimodal treatment approach.

## 2 Case report

A 66-year-old insulin-dependent diabetic Caucasian man who resided in a subtropical climate presented for eye pain in his right eye (OD) without a history of obvious trauma. He had an ocular history of herpes simplex virus (HSV) with subsequent neurotrophic keratopathy status post 20% temporal tarsorrhaphy. Additionally, he had a corneal ulcer (culture positive for *Candida albicans* and *Candida parapsilosis)* that responded to topical amphotericin B and voriconazole which had healed 3.5 months prior. This was quiescent when an HSV-related dendrite occurred 2 months later, so the patient had been continued on topical amphotericin B twice daily and oral valacyclovir three times daily prophylactically. Just before presenting with the current findings, topical prednisolone had been used for 2 weeks to treat residual corneal haze and inflammation after resolution of the HSV keratitis.

**Fig. 1:**
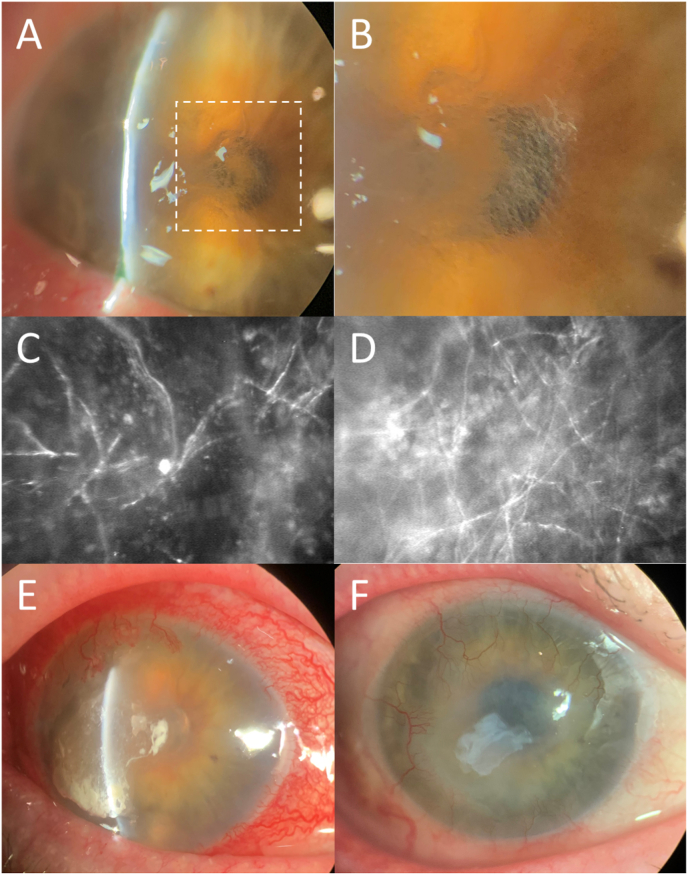
Slit lamp photograph demonstrating corneal ulcer with surrounding anterior stromal haze, thinning, and hair-like, filamentous elements on retroillumination (1A & 1B). Confocal microscopy showed stromal structures suspicious for filamentous fungal species (1C & 1D). Slit lamp photograph demonstrating worsening injection, ulcer size, edema, and neovascularization despite topical and oral antifungal treatment (1E). Resolution following superficial keratectomy with placement of layered amniotic membrane, intrastromal and subconjunctival antifungal injections, and a tarsorrhaphy; note the residual amniotic membrane filling the previously thinnest areas (1F).

On examination, visual acuity was 20/400 OD and 20/25 in the left eye (OS). The cornea demonstrated scarring and thinning from his prior HSV and fungal infections. There were two epithelial defects, 1.5 × 1.0 mm and 1.0 × 2.5 mm in size, with surrounding anterior stromal haze and hair-like, filamentous elements on retroillumination (Fig. 1A and B). Cultures from corneal scrapings were obtained, and confocal microscopy showed stromal structures suspicious for filamentous fungal species (Fig. 1C and D). A smear did not reveal any organisms or fungal elements. Topical prednisolone acetate and amphotericin B were discontinued, and the patient was started on topical natamycin, topical voriconazole, oral fluconazole 200 mg daily, and prophylactic topical besifloxacin. The epithelial defect progressed over the next two weeks (no change on confocal microscopy, Fig. 1E) and the patient admitted nonadherence to the high frequency of antifungal drops. The decision was made to supplement with voriconazole intrastromal and subconjunctival injections two to three times weekly. There was slow improvement in the ulcer size. The topical natamycin was poorly tolerated and had not shown efficacy early in the clinical course, so therapy was switched to topical and oral posaconazole (in place of oral fluconazole). Topical posaconazole (off-label use) was not tolerated due to burning pain upon administration.

**Fig. 2:**
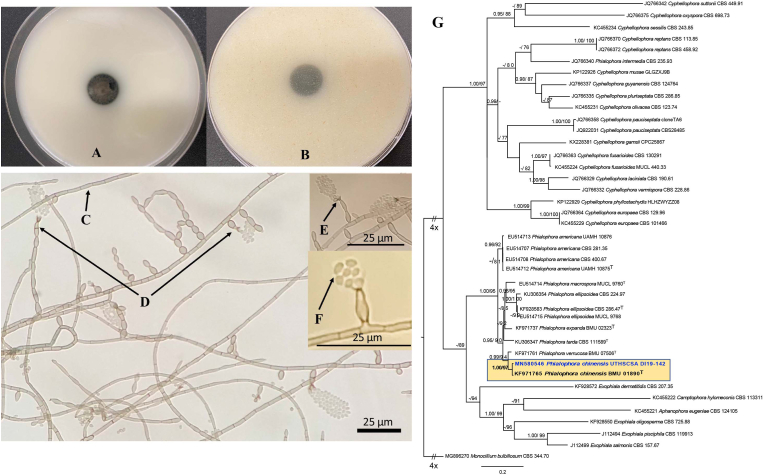
Macroscopic and microscopic features of *Phialophora chinensis* UTHSCSA DI19-142. Colonies (woolly, pale gray to dark olivaceous [olive-brown]) at 25 °C (no growth at 40 °C), 15d, left to right: obverse and reverse on OA (2A & 2B). Light micrographs at 63 days: 2C. hyphae (brown hyphae, regularly septate), 2D. phialides (smooth walled, sessile or at the tip of occasionally septate catenulate cells), 2E. collarettes (widely flaring in the broadest part, with distinct periclinal thickening), and 2F. conidia (globose to subglobose). 2G. The best scoring maximum likelihood tree based on β-tubulin gene (TUBB). Bayesian posterior probability (PP) values (≥95, Left) and maximum likelihood bootstrap values (≥75, Right) are shown at the nodes. Lower values of support are indicated with a hyphen. T = type strains. GenBank accession numbers are located before the species names and culture collection accession numbers are located after the species names. *Monocillium bulbillosum* CBS 344.70 was selected as outgroup. (For interpretation of the references to colour in this figure legend, the reader is referred to the Web version of this article.)

After 1.5 months, the initial culture returned positive for mold (2 additional cultures had been taken without any growth over this period). The fungus was sent to the Fungus Testing Laboratory (FTL) at the University of Texas Health Science Center at San Antonio for identification and susceptibility testing and was identified as *Phialophora chinensis* (Fig. 2A–G)*.* After a literature search noted promising results in treating a recalcitrant *P. verrucosa* keratitis infection with corneal cross-linking (CXL),^2^ this was performed for our patient. Following CXL treatment (Dresden protocol),^10^ there was a clinical improvement, and topical and oral antifungals were maintained. Confocal microscopy appeared clear of fungal elements at this point. The patient was also treated with a cryopreserved amniotic membrane (Prokera, Biotissue) and a course of topical cenegermin-bkbj 0.002% (Oxervate, Dompe) given the neurotrophic appearance (Stage 3, small remaining epithelial defect) and decreased corneal sensitivity. While there was initial improvement with these treatments, the epithelial defect eventually worsened over the course of two months. Confocal microscopy demonstrated minimal remaining fungal elements, so voriconazole intrastromal and subconjunctival injections were resumed. Another culture and smear from corneal scrapings revealed no organisms or growth. Superficial keratectomy with placement of layered amniotic membrane was performed, along with intrastromal and subconjunctival antifungal injections (amphotericin B and voriconazole), and a tarsorrhaphy (lateral, increased to 40%). The epithelial defect healed (Fig. 1F), and repeat confocal microscopy confirmed no evidence of fungal elements. Oral antifungals were discontinued after 6 months. Laboratory follow-up was performed periodically to monitor renal and hepatic function (renal more frequently checked due to concomitant valacyclovir).

**Fig. 3:**
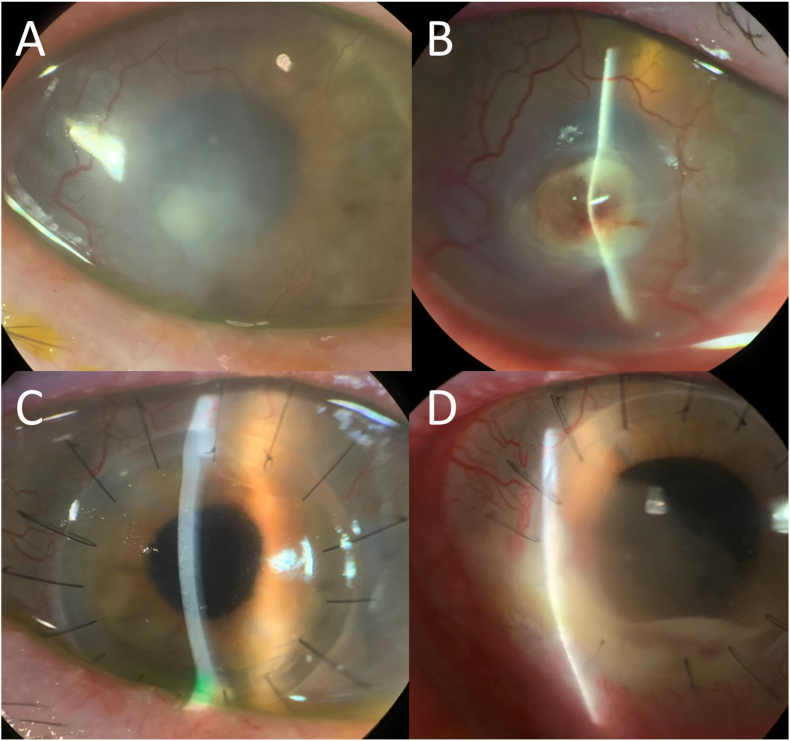
Slit lamp photograph demonstrating recurrence of a new intrastromal infiltrate that appeared after 9 months of quiescence (4A). Despite topical, oral, and intrastromal antifungals, there was rapid worsening and perforation (4B). Hemorrhage and hyphema can be noted as the patient also sustained a blunt trauma during this period. A clear penetrating keratoplasty was maintained for more than 6 months (4C), until the patient suddenly presented with a perforated ulcer at the graft periphery (4D). The infiltrate appearances of the recurrences did not have the same filamentous appearance as the initial presentation.

**Fig. 4:**
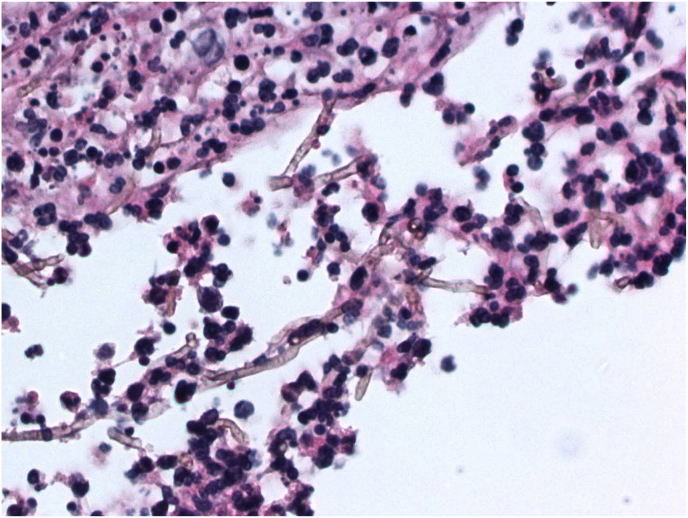
Corneal specimen from our patient's penetrating keratoplasty demonstrating septate hyphae with rare right-angle branching and associated pustular necrosis (Periodic acid-Schiff, X400). Acknowledgement: Dr. Marc Silverberg.

Slit lamp and confocal examination were clear for 9 months, when a new intrastromal infiltrate appeared (Fig. 3A and B). No topical corticosteroids had been used, but the patient had received a corticosteroid injection for his knee a few weeks prior. Voriconazole drops had been maintained. Despite restarting topical amphotericin and intrastromal injections (both voriconazole and amphotericin), there was rapid worsening and perforation. Penetrating keratoplasty (PK) was performed with subsequent vitrectomy (with intravitreal voriconazole) 2 days later for endophthalmitis. Pathologic evaluation of the PK specimen revealed septate hyphae with rare right-angle branching, and few nonbudding yeast forms (Fig. 4). Topical corticosteroids were held for about 1 month (although on cyclosporine 0.05% twice daily), while intravitreal and intracameral antifungal injections were continued during that period. Topical prednisolone was resumed with continued voriconazole at the recommendation from our retina colleagues to decrease inflammation, and the patient's PK graft remained quiet and clear for 6 months with best uncorrected visual acuity 20/100 (Fig. 3C). The patient returned at this time with a new infiltrate at the graft-host-junction (GHJ) with a perforation (Fig. 3D). A larger eccentric PK was performed to encompass this area with cryotherapy and antifungal injections applied to the adjacent cornea and sclera. Microbiology results from both PK specimens were identified as *Phialophora chinensis.* A small endoplaque and infiltrate developed along the GHJ but remained stable with intracameral and intravitreal voriconazole and amphotericin injections. Unfortunately, the patient was lost to follow-up for 2 months due to a hospitalization at an outside facility for altered mental status and multi-organ failure. When care resumed, corneal findings were diminished by the systemic corticosteroids the outside hospital had started. Despite initial worsening after corticosteroids were discontinued, weekly to twice weekly intracameral and intravitreal amphotericin injections in the setting of systemic posaconazole stabilized the infiltrate/endoplaque. Renal and hepatic function were followed closely by the medicine team. Due to a diagnosis of metastatic pulmonary malignancy, the patient was placed on hospice and lost to follow-up.

## 3 Fungal culture and susceptibility testing

The isolate (UTHSCSA DI19-142) was subcultured onto potato flakes agar (PFA) and incubated at various temperatures (25 - 40 °C). Growth was also assessed on oatmeal agar (OA). Colonial features were examined after 14–21 days of incubation. DNA sequencing and phylogenetic analysis are described below. Susceptibility testing was performed according to the CLSI M38 reference standard.^11^

## 4 Phylogenetic assessment

Mycelial mass from the isolate was harvested from PFA for DNA extraction, and genomic DNA was extracted following previously described protocols.^12^ The ITS rDNA region (ITS), the D1 and D2 domains of the large subunit rDNA (LSU) and partial beta tubulin (TUBB) genes were amplified and sequenced to compare with sequences of the same loci in previous studies.^9^^,^^13^ PCR and sequencing were carried out using primer pairs BMBC-R and NL4R (https://sites.duke.edu/vilgalyslab/rdna_primers_for_fungi/) for ITS, LSU,^14^ BT2a and Bt2b for TUBB,^15^ and BLASTn searches were performed. Based on BLASTn results, phylogenetic analysis was conducted for TUBB to assess the relationship of our isolate to members of the *Phialophora verrucosa* complex. Sequences were aligned using MUSCLE as implemented in Sequencher ver 5.4.6 build 46289 (Gene Codes Corp. Ann Arbor, MI, USA).^16^ Phylogenetic analysis using the maximum likelihood method and bootstrap were conducted in IQ-TREE^17^ and Bayesian inference was conducted in MrBayes v3.2.5.^18^

## 5 Identification results

Macroscopic and microscopic examination showed phenotypic characteristics salient with *Phialophora* species (Fig. 2A–F). The ITS, partial LSU and partial TUBB gene were chosen to compare with sequences of the same loci in previous studies.^9^^,^^13^ The DNA sequences) were used to perform BLASTn searches in GenBank (https://www.ncbi.nlm.nih.gov/) (GenBank accessions numbers shown in Table 1) as well as in Mycobank (https://www.mycobank.org/page/Pairwise_alignment). As these results were inconclusive, phylogenetic analysis of the partial TUBB gene was performed and this showed the isolate was conspecific to *P. chinensis* (Fig. 2G). Minimum inhibitory concentrations (MICs) against amphotericin B, fluconazole, natamycin, posaconazole and voriconazole are shown in Table 1.

**Table 1:** Results of GenBank BLASTn percent matches to type isolates and antifungal susceptibility testing. Percent matches to other isolates for ITS and LSU using DNA sequence databases available in Mycobank are shown in parenthesis.

Species	ITS	LSU	TUBB
*P. chinensis* BMU01890^T^	98.98%	N/A	99.12%
(*P. chinensis* BMU07622)			
(*P. macrospora* MUCL15541)	(99.83%)	(99.84%)	
*P. verrucose* BMU07506^T^	98.81%	99.81%	96.05%
(*P. americana* CDC-B2152)			
(*P. chinensis* CNRMA18.587)	(99.68%)	(99.83%)	
*P. ellipsoidea* MUCL 9768^T^	99.67%	99.67%	91.67%
(*P. chinensis* BMU07609)			
(*P. americana* CBS840.69)		(99.82%)	
*P. macrospora* MUCL 9760^T^	97.84%	99.84%	90.61%
(*P. chinensis* BMU07613)			
(*P. americana* MUCL 39979)		(99.67%)	
GenBank Accession No. for UTHSCSA DI19-142	MN381348	MN381366	MN580546

**Antifungal Susceptibility**	**Minimum inhibitory concentration (μg/ml)**		
Amphotericin B	0.5		
Caspofungin	0.125		
Fluconazole	8		
Natamycin	2		
Posaconazole	≤0.03		
Voriconazole	0.125		

## 6 Discussion

The family *Herpotrichiellaceae* (*Chaetothyriales*), of which the type species is *Phialophora verrucosa,* includes several asexual species classified in the genera *Cladophialophora, Exophiala* and *Fonsecaea*. Molecular typing using restriction fragment length polymorphisms (RFLP) indicated that strains identified as *P. verrucosa* are genetically variable and are considered a complex.^19^ Li et al. conducted a phylogenetic, morphologic, physiologic and ecological reassessment of the *P. verrucosa* complex resulting in the identification of four new species, which include *P. chinensis*, *P. ellipsoidea*, *P. expanda* and *P. tarda*.^9^

Mycotic keratitis is encountered more frequently in immunocompromised hosts, such as transplant recipients, AIDS patients, and diabetics.^20^ Local predisposing factors include trauma, especially with vegetable matter, contact lens wear, topical corticosteroid use, and ocular surface disorders. Predisposing factors for our patient included an ocular history of HSV keratitis, neurotrophic keratopathy, prior fungal ulcer, subtropical residence, recent topical corticosteroids in the setting of diabetes (well-controlled at the time of this illness, but previously with poor control, as evidenced by ongoing peripheral vascular complications) and an undiagnosed malignancy. Another challenging aspect to treating this refractory case was the patient's nonadherence to the treatment regimen (especially early in the course) and difficulty in maintaining close follow-up.

Although dematiaceous fungi have been reported to be the third most common cause of fungal keratitis behind Aspergillus and Fusarium species, the causative organism in our patient, *P. chinensis*, has not previously been reported as a cause of keratitis. Nearly all known strains of *P. chinensis* are environmental, mostly isolated from soil and plant debris, and display opportunistic behavior after local trauma.^1^ Two of the strains examined in the study by Li et al. were derived from chromoblastomycosis skin lesions and revealed colonies of moderately slow growth that were olivaceous and black, with a pale olivaceous center. *P. chinensis* produces thick-walled, swollen cells strongly resembling the muriform cells of chromoblastomycosis and some yeast-like cells with scant hyphae. These features comprise its virulence factors (ie. thick cell walls, muriform cells, and yeast-like phases), which exert variable influence depending on the location and severity of the infection.^9^ No previous literature has been found on mycotic keratitis caused by *P. chinensis*, although several cases have been reported involving its closely related species *P. verrucosa*. Because of the chronic and relapsing nature of *Phialophora* infections, there is typically a progressive course despite aggressive antifungal therapies that often leads to corneal transplantation. In the first described case, Wilson et al. showed resistance of *P. verrucosa* to amphotericin B and itraconazole but susceptibility to ketoconazole.^6^ Taechajongjintana et al. showed no response of *Phialophora* to amphotericin B, natamycin, or itraconazole, but susceptibility to voriconazole.^2^ In contrast, Banitt et al. reported no activity of voriconazole, fluconazole, or amphotericin against P. verrucosa. ^21^ Hirst et al. only eradicated the *Phialophora* infection after penetrating keratoplasty due to clinical progression despite aggressive topical and systemic antifungal treatment.^3^^,^^22^

Our case of *P. chinensis* did not respond clinically to topical antifungals (including natamycin, voriconazole, amphotericin B) with concurrent oral antifungal therapy (i.e., fluconazole or posaconazole). *In vitro* susceptibility results demonstrated the most potent activity for posaconazole and voriconazole; however, topical concentrations may not have been high enough to inhibit this particular fungus. Another consideration includes our patient's relative noncompliance regarding the frequency of the topical therapy. The best response was frequent intrastromal antifungal injections with adjunct CXL. Concomitant treatment of the neurotrophic keratopathy was crucial to heal the epithelial defect and minimize access of residual fungus to the stroma. This case also highlights the utility of confocal microscopy to gauge response to therapy.

While we are unclear where this infection originated, we believe the patient's residence in a subtropical climate in the setting of a compromised ocular surface were a setup for infection. Besides normal exposure to the outdoors in this locale, the patient had also noticed dark mold growing in his pair of swimming goggles. This was cultured but did not yield any results. Although not endorsed on presentation, patient did demonstrate a tendency to periodically scratch/injure his eye (with his finger or other objects) which may have contributed. Unfortunately, there were either two recurrences or reinfections with the same fungus after a period of quiescence. These episodes occurred despite continued antifungal prophylaxis with oral and/or topical medications. The appearance of an intrastromal infiltrate after the first period of quiescence may argue for recurrence. Both instances had a much more rapid course than the initial presentation, quickly leading to perforation and necessitating surgical intervention.

## 7 Conclusions

Keratitis caused by dematiaceous fungi can be variable in clinical presentation and course. Infections can lead to both superficial and deep fungal ulcers and demonstrate an aggressive, chronic course potentially leading to corneal perforation. Despite a multimodal treatment regimen, this case demonstrates the recalcitrant and potentially recurrent nature of *Phialophora chinensis* fungal keratitis.

## Funding

The authors did not receive funding for any of this work.

## Authorship

All authors attest that they meet the current ICMJE criteria for Authorship.

## Consent

Consent to publish the case report was not obtained. This report does not contain any personal information that could lead to the identification of the patient.

## Declaration of competing interest

There are no relevant disclosures. AYC has consulted for LayerBio. JDS reports consulting for Allergan, AbbVie, Alcon, Aldeyra Therapeutics, Bausch & Lomb, BioTissue, ClarisBio, Dompe, EyeDetec, Eye Point, EyeGate, Fortress Bio, NovaBay, Novartis, Noveome, LayerBio, Mallinckrodt, Mati, Ocular Therapeutix, Kala, RPS, Tarsier, Tearlab, Johnson & Johnson, Fidia, Clarios, Visus, Topivert, Noveome, Oyster Point, Santen, Sun Pharmaceutical Industries, Eyevance, ScienceBased Health, and Quidel; and ownership interest in ClarisBio, Noveome, EyeDetec, EyeRx Pharma, Oyster Point, RPS, TearLab, EyeGate, Strathspey Crowne, Mati, and CVP Partners. The following authors have no financial disclosures: BP, CFCG, CS, NPW, RD.
